# Case Report: An isolated medial condylar fracture of the distal humerus in an adolescent baseball pitcher

**DOI:** 10.3389/fsurg.2026.1853523

**Published:** 2026-07-13

**Authors:** Shih-Feng Hung, Chih-Yuan Jen, Yu-Pao Hsu, Ming-Te Cheng, Yu-Hsu Chen

**Affiliations:** 1 Department of Orthopedic Surgery, Taoyuan General Hospital, Ministry of Health and Welfare, Taoyuan, Taiwan; 2 Department of Surgery, Taoyuan General Hospital, Ministry of Health and Welfare, Taoyuan, Taiwan

**Keywords:** adolescent injury, distal humerus, isolated medial condylar fracture, pediatric fracture, thrower's fracture, throwing motion

## Abstract

**Background:**

Thrower's fracture, a non-traumatic humerus fracture related to the throwing motion, has been reported in a few studies in the literature. To the authors’ knowledge, there has been no report on an isolated medial condylar fracture of the distal humerus induced by the throwing motion to date. In this study, we provide a potential explanation to the pathomechanism of such a rare type of thrower's fracture.

**Case presentation:**

We present a rare case of a 17-year-old Asian adolescent male who suffered an isolated medial condylar fracture of the distal humerus after baseball pitching. A right elbow X-ray showed a displaced fracture of the right medial humeral condyle running through the trochlear groove. Open reduction and internal fixation with plating were performed smoothly and the patient was discharged in a stable condition. The range of motion of the elbow was intact and the boy returned to sports activities without any complications.

**Conclusions:**

This was the first study that showed the throwing motion may induce isolated medial condylar fractures of the distal humerus. The potential pathomechanism of this injury was elucidated by drawing upon the current understanding of the mechanisms involved in various components of complex injuries.

## Background

Most humerus fractures result from direct traumatic events. Thrower's fracture, a non-traumatic humerus fracture related to the throwing motion, has been reported in a few studies in the literature. This type of fracture is thought to result from torsional forces exerted by muscles attached to the humerus during the intricate throwing motion (1, 2). The act of throwing is a complex motion wherein various muscle groups interact synergistically and antagonistically, exerting forces on the humerus. Although thrower's fracture was already reported to be an uncommon type of fracture in adolescents, to the authors’ knowledge, there has been no report on an isolated medial condylar fracture of the distal humerus induced by the throwing motion to date. In this study, we provide a potential explanation to the pathomechanism of such a rare type of thrower's fracture.

## Case presentation

**Figure 1:**
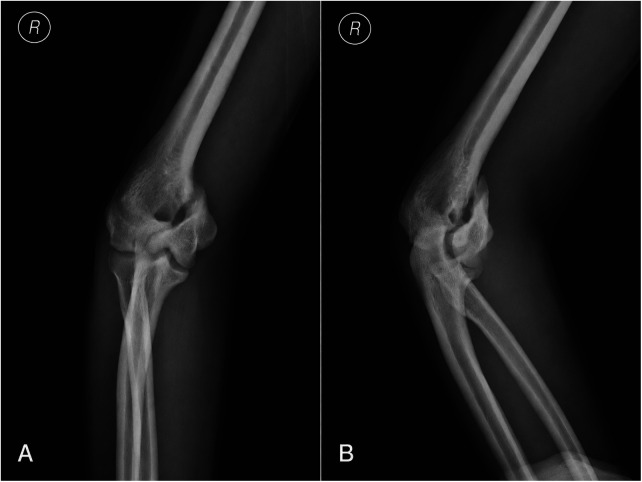
**(A)** An anterior-posterior (AP) view of the right elbow of the patient in the emergency room. **(B)** A lateral view of the right elbow of the patient in the emergency room.

A 17-year-old Asian adolescent male was admitted to the emergency room of our hospital because of severe right elbow pain that developed immediately after baseball pitching during a tryout for a college varsity team earlier during the day. The patient denied any systemic underlying and traumatic injury. He had been playing on the varsity baseball team for his high school. He did not warm up prior to pitching and was a starting pitcher 2 days prior to the incident. The initial physical examination disclosed swelling, ecchymosis, and tenderness to the medial side of the right elbow. The accompanying symptoms included limited mobility and range of motion (ROM) in flexion and extension. The distal neurovascular status of the right arm was intact without a claw hand. A right elbow X-ray showed a displaced fracture of the right medial humeral condyle running through the trochlear groove (AO/OTA 13B2.1) (Figures 1A, B). The patient then received open reduction internal fixation the next day.

### Operation

**Figure 2:**
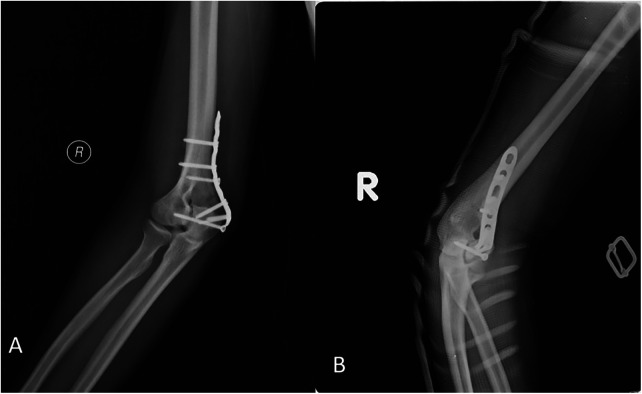
**(A)** A postoperative AP view of the right elbow of the patient. **(B)** A postoperative lateral view of the right elbow of the patient.

Open reduction was done through a direct medial approach under general anesthesia. The first step was to identify the ulnar nerve. The ulnar nerve was carefully identified and protected throughout the procedure. Gentle neurolysis and release were performed to avoid traction or compression during fracture reduction and plate fixation. The fracture fragment was exposed and reduced using a 3.5 mm AO distal humeral locking compression plate in the accurate position. A long-arm splint was then applied. A postoperative X-ray of the right elbow was taken (Figures 2A, B). The patient was evaluated after anesthesia recovery; distal circulation, sensory function, and motor function were intact. He was discharged from our hospital in a stable condition.

### Follow-up

**Figure 3:**
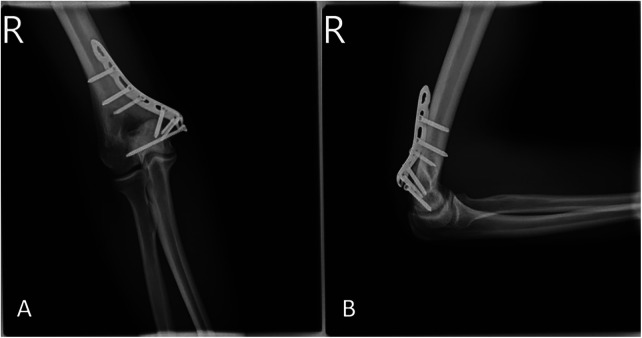
**(A)** A 3-month postoperative follow-up of an AP view of the right elbow of the patient. **(B)** A 3-month postoperative follow-up of a lateral view of the right elbow of the patient.

**Figure 4:**
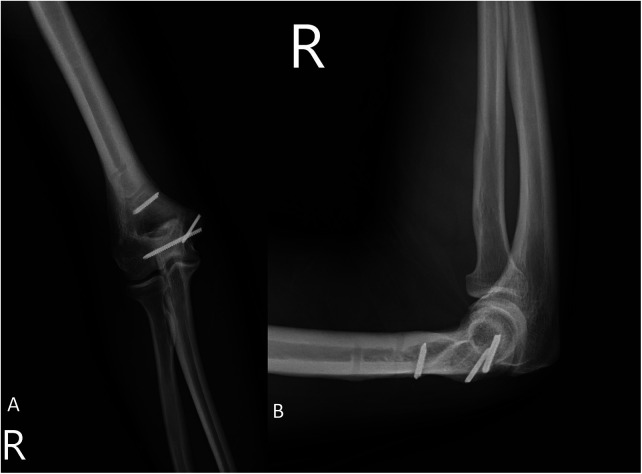
**(A)** A 2-year postoperative follow-up of an AP view of the right elbow of the patient after the implant was removed. **(B)** A 2-year postoperative follow-up of a lateral view of the right elbow of the patient after the implant was removed.

The patient visited the outpatient clinic a week after the operation for a follow-up. The posterior splint was removed and converted to a sling. Active rehabilitation and ROM exercise were encouraged. At the 3-month follow-up, the boy regained full strength and ROM in the right elbow. An X-ray revealed complete bone healing of the medial condyle of the right humerus (Figures 3A, B). The patient was lost to follow-up for 2 years before he returned to the clinic because of a protruding implant irritation. A surgery was performed and the plate was removed; however, two broken screws were noted and left inside the bone (Figures 4A, B). The ROM of the elbow was intact and no specific discomfort was felt by the patient. He was able to return to sports activities without any complications.

## Discussion

Wilmoth et al. reported the first case of a thrower's fracture in 1930. Although the highest incidence has been noted in overhand throwing sports such as baseball and softball, other activities related to throwing, such as grenades, snowballs, shot puts, dodge balls, cricket balls, and handballs, have all been documented (2). Fractures resulting from these activities typically exhibit a spiral pattern, extending from the midshaft to the distal shaft of the humerus (3). To the authors’ knowledge, there has not been any case report to date on an isolated medial condylar fracture of the distal humerus in adolescents injured by baseball throwing.

Fractures related to throwing can result from an underlying weakness in the bone structure of the humerus, stemming from mechanical, physiological, or pathological factors (4). Occasional pitching by recreational athletes has been associated with humeral thrower's fractures due to inadequate bone remodeling, which typically strengthens bones in more consistently active and professional pitchers (5). The adaptation of the pediatric humerus to throwing forces is notably less robust than that of the adult humerus to throwing forces. In our case, the patient was a high-school athelete who had been training relentlessly prior to his injury. Osteochondritis dissecans and uncoordinated muscular contraction due to fatigue might have contributed to the injury.

The throwing motion is considered a process involving a kinetic chain of elements that functions to optimize the efficiency of the proximal segments and decrease force loads seen at smaller, distal segments such as the elbow. Weakness, imbalance, or stiffness within the kinetic chain can cause a risk of elbow injury. The throwing motion has been classified into five stages: wind-up, early cocking, late cocking, acceleration, and deceleration with follow-through. The significant forces and twists produced during the subsequent three stages (late cocking, acceleration, and deceleration) are responsible for most pitching-related injuries (6, 7).

During the late cocking phase, what 's most noticeable is the intense level of shoulder external rotation achieved as the torso finishes its twist. Dillman and his team noted that the external and internal rotation in pitching was among the most dynamic actions within the human body (8). After the foot lands, the arm rotates externally across a span of 125°, moving from approximately 50° of external rotation upon foot contact to roughly 175° at its peak external rotation. Different studies have identified the maximum shoulder external rotation in professional baseball pitchers to be between 160° and 185°. This pronounced external rotation enables the pitcher to exert a speeding force to the ball over the longest potential distance in the following acceleration stage of the pitch. In our case, the combination of external rotation of the humerus, rapid extension of the elbow by extensor muscles, and the muscular valgus torque by twisting of the flexor muscles and pronator teres of the forearm during the throwing motion might be the potential mechanism of the injury.

In addition to torsional and valgus stress during the late cocking and acceleration phases, another possible mechanism is the vertical shear force across the ulnohumeral articulation. Rapid elbow extension and force transmission through the trochlea during pitching may create a vertical cutting effect on the medial condyle, contributing to the observed fracture configuration.

Fractures of the medial condyle are susceptible to complications such as non-union, growth plate cessation, joint line irregularity, and deformity. To avert these issues, timely detection, proper realignment, and secure fixation are essential. Reported non-union rates for medial condylar fractures vary from 7.4% to 33.3% (9–11). Some experts advise against surgery because of potential complications. Certain studies, however, suggest that treating non-union through osteotomy or osteosynthesis can result in joint rigidity and discomfort, while others have indicated positive outcomes (12, 13). In our case, non-union wasn't an observed complication after open reduction and internal fixation with a locking plate were applied.

## Conclusion

This study was the first that demonstrated the throwing motion may induce an isolated medial condylar fracture of the distal humerus. The potential pathomechanism of this injury was elucidated by drawing upon the current understanding of the mechanisms involved in various components of complex injuries. Assessing athletes for stress fractures and recommending activity adjustments for those showing early signs of arm pain during throwing could potentially lower the occurrence of humerus fractures related to throwing.

## Data Availability

The original contributions presented in the study are included in the article/Supplementary Material, and further inquiries can be directed to the corresponding author.
